# Reviewers acknowledgement

**DOI:** 10.1002/2211-5463.12756

**Published:** 2019-12-02

The Editors of *FEBS Open Bio* would like to thank all those who have given their time and expertise to review articles submitted for publication in Volume 9. Although *FEBS Open Bio* does not seek to judge the importance of submissions, our reviewers carefully scrutinize the experimental design and results of all papers and challenge authors about their conclusions.

The names of these reviewers are listed below; we apologize if we have inadvertently omitted anyone.

Heba Abdella

Luis Alberto Acuña‐Amador

Kenneth William Adams

Wilfried Admiraal

Yukio Ago

Jinwoo Ahn

Masanori Aikawa

Yusuf Akhter

Ozan Akkus

Khondoker Akram

Virginia Helena Albarracín

Marcos R Alborghetti

Miguel Alejandre Alcazar

Arshad Ali

Isabel Alicia Luthy

Malcolm Alison

L A Allison

Francisco Altamirano

Xu An

Ruchi Anand

Michael Antosh

Kazutaka Araki

David H Ardell

Javier Arsuaga

Higinio Arzate

Avraham Ashkenazi

Ianire Astobiza

Mohammad Athar

Shannon Wing‐NgorAu

Benjamin Audit

Sylvie Babajko

Leila Bagheri

Gang Bai

Manoj Baranwal

Joey V Barnett

Marco Baroni

Eliaz Barquero

Elias Barquero

Reetobrata Basu

Claude Becker

Sven‐Erik Behrens

Alexandre Benedetto

Stefania Bertora

Tushar Beuria

Eric Beyer

Wilbert Bitter

Philippe Blache

C Blenkiron

Todd Blevins

Luana Pereira Borba‐Santos

Eric Bortz

Marc Boutry

Caroline Bowsher

Kristen Boyle

Peter J Bradley

Marta Helena Branquinha

Gustavo Bressan

Vania Brissos

Anke Burmester

Zachary Burton

Verónica A Burzio

Jinquan Cai

Ye‐Jun Cai

Hua Cao

Salvatore J Joseph Caradonna

Teresa Cardoso

Christopher P Cardozo

Daniela Carlisi

Giorgio Caserta

Rui Castro

Silver Ceballos

Valentina Celestini

Dhyan Chandra

R Jeffrey Chang

Max Chavarria

Jin‐Ran Chen

Dong Chen

Xuqiao Chen

Yu Chen

Xin Chen

G Chen

Wei Chen

Liyang Chen

Chung‐Hwan Chen

Yaodong Chen

Tsung‐Lin Cheng

Joyce Chiu

Vinay Choubey

Cheng‐Ming Chuong

Rachel Clifford

Sebastien Corre

Alessandro Costa

James Costello

Robert R Crichton

Richard Cubbon

Liza Cubeddu

Aurora Daniele

Matthew Daniels

Marie Davídková

John Davies

Claudia M d'Avila‐Levy

Malgorzata Dawidowska

Bart De Geest

Carsten Deppermann

Jeremy P Derrick

Mahesh Desai

Kishor Devalaraja‐Narashimha

Harpal Dhillon

Maria Teresa Di Martino

Giuseppina Di Stefano

Pietro Didazio

Vittoria Disciglio

Francesco Dituri

Klaus Dornmair

Leonardo dos Reis Silveira

Ian Dransfield

Mulong Du

Shiwei Duan

E Dundar

M Beatriz Durán‐Alonso

Clive Ellory

Michael J Emanuele

Edward Emmott

Motomi Enomoto‐Iwamoto

Winnie Eskild

Frank O Fackelmayer

Chengming Fan

Chao Fang

Candida Fasano

Yingang Feng

Julian Fernandez

Gerardo Ferrer‐Sueta

Céu Figueiredo

Andrew Filby

Emily Flashman

Biff Forbush

Florentia Fostira

Salvatore Foti

Kristin French

Li Fu

Liyun Fu

Lin Fu

Hans‐Lothar Fuchsbauer

Ko Fujimori

Masanori Fukazawa

Ryuya Fukunaga

Wataru Fukuokaya

Mariam Gachechiladze

Laura García Bermejo

Katja Gehmlich

Andrea Geisz

Wilfred Germeraad

Sandra E Ghayad

Pegah Ghoraeian

John Glover

Isabel Gómez

Mehmet Gonen

Weijuan Gong

Sven‐Ulrik Gorr

Mark D Gorrell

Caroline Gorvin

Wieslawa Grajkowska

Angelica Granados‐López

Mohammed E Grawish

Michael Greene

Jenna Gregory

Darren Griffin

Valentina Grossi

K Grzelkowska‐Kowalczyk

Xinbin Gu

Jinghui Guo

Yunxue Guo

George Haddad

Shuhua Han

Talita Hanchuk

Hidetaka Hara

Jennifer Hardingham

Martin Haslbeck

Zhaohui He

Kenneth R Hess

Rainer L Heuchel

Jan Heyda

Joseph Ho

Philip J Hogg

Tomoyuki Honda

Rüdiger Horstkorte

Yanling Hu

Guoxin Hu

Chang‐Ming Huang

Jiaquan Huang

Ying Huang

Kun Huang

Koichi Iijima

Laertis Ikonomou

Andrea Ilari

Diana Imhof

Javier Inserte

Osamu Ishibashi

Katsuhiko Ishihara

Tetsuro Ishii

Akira Ishisaki

Tohru Itoh

Masahiro Iwamoto

Yasuhiko Izumi

Chathuraka T Jayasuriya

Marc G Jeschke

Niels Jessen

Peng Ji

Tongmeng Jiang

Bin Jiang

Zongliang Jiang

Xiancheng Jiang

Fengliang Jin

Xin Jin

Maria João Prata

Mark S Johnson

Muhammad Junaid

Alexander Kalachev

Murugan Kalimutho

Eniko Kallay

George D Kalliolias

Tomasz Kantyka

Koichi Kato

M Katoh

Gurmeet Kaur

Savneet Kaur

Bodil Kavli

Istvan Kenessey

M Khajehpour

Bilon Khambu

Shafiq A Khan

Alex Kharitonenkov

Alexei Kharitonenov

Takahiro Kikawada

Hyong Kyu Kim

Keekwang Kim

Young Ho Kim

Jeongwoo Kim

Yohei Kirino

Kunal Kishor

Hisataka Kitano

Gerhard Klebe

Jakob G Knudsen

Yasuhiro Kobayashi

Peppi Koivunen

Jeremy Koppel

HK Koul

Yutaka Koyama

Katarzyna Koziak

Paweł Kozielewicz

Makiko Kumagai‐Braesch

George Kyriazis

Peter Lackie

Paula Lam

Noelia Lander

Mattia Lauriola

José Luis Lavín

Maryse Lebrun

Po‐Shun Lee

Jason Lee

Chu‐Hee Lee

Young‐Ho Lee

H K Leiros

Roger Leng

Martina Lepore Signorile

Trond P Leren

Mark P Lewis

Lei Li

Minqi Li

Yong Li

Yirong Li

Mingchuan Li

Chunwei Li

Po‐Huang Liang

Xiaofeng Liao

David Liebner

Rebecca Lim

Chieh‐Yu Lin

Wen‐Ge Liu

Marco Lolli

Donald Loo

César Lopez‐Camarillo

David Loria

Rik Lories

Hua Lou

Edward Lowe

Kun Lu

Qingchun Lu

Giuseppe Lucarelli

Christopher A MacRaild

Mattias Magnusson

Hanns‐Christian Mahler

František Marec

Peter Marhavy

Catalin Marian

Enrique Martinez‐Force

Simin Masoudi

David Masson

Takaaki Masuda

Yuriko Matsuzaki

Konstantinos Mavridis

Jennifer A Maynard

Kimberly McCall

Clara McCarthy

Frances Meier Gibbons

Raquel Mejias‐Luque

Maria Mempin

Tony Merriman

Akira Mima

Hamid Reza Mirzaei

Mineyuki Mizuguchi

Ahmed Mohamed

Athar Mohammad

Pooi Ling Mok

Nadia Monesi

Carmen Montesinos

Paula Mulo

Karthikeyan Muthusamy

Terence Myckatyn

Shigeyuki Nada

Soheil Naderi

Taku Nagai

László Nagy

Javid Nahand

Firzan Nainu

Tetsuji Naka

Atsushi Nakagawa

Tsukasa Nakamura

Takashi Nakamura

Masayoshi Nakamura

Eijiro Nakaruma

Derek Narendra

Heinz Peter Nasheuer

Rosa Navarro

Nouri Neamati

Hovav Nechushtan

Mark Nelson

V V Nenasheva

Joseph Nickels Jr

Guoping Niu

Josephine Nocillado

Nicholas Noinaj

Saori Nonaka

Petr Novák

László Nyitray

Rossana Occhipinti

Shigeru Okada

Valentyn Oksenych

Fumihiko Okumura

Mitsuhiko Osaki

Ann‐Kristin Östlund Farrants

Motoyuki Otsuka

T Z Parris

Iva Pashkuleva

Evgeny V Pavlov

Haiping Pei

Patrik Persson

Alessia Peserico

Maire Peters

A Petry

Mike Philpott

Sebastien Pichoff

Matthew Picklo

Giuseppe Pignataro

Rameshe Pillai

Adrian Pinto‐Tomas

Katie Podshivalova

Indira D Pokkunuri

Richard K Porter

Monica Prado

Aruna Prakash

Bo Qin

Vanessa Ramirez

Eric Gustavo Ramírez‐Salazar

Qitao Ran

Oliver Rando

Catarina Raposo Dias Carneiro

Angela Raucci

Prashanth Rawla

Aravind Reddy

Patrick Reinke

Eduardo Reis

Solveig Reitan

Edyta Reszka

N Rhaleb

Eduardo Rial

Jeremy N Rich

Selena Richards

Samantha J Richardson

Dean Ripple

Federica Riva

Remy Robert

Josias Rodrigues

J L Rodrigues‐Fernandes

Claudina Amelia Rodrigues‐Pousada

Javier Rodriguez Villanueva

Tatiana K Rostovtseva

Barak Rotblat

Pawel Rubis

Margherita Ruoppolo

Maja Sabol

Juan CarlosSaez

Takashi Saito

Yoichi Sakakibara

Luca Sala

Valentina Sala

Helioswilton Sales‐Campos

Adrian Sanchez‐Fernandez

Isabel Sanchez‐Perez

Prasanna Kumar Santhekadur

Mario Saparrat

Lígia M Saraiva

Pratik Satya

Michael Scharl

Laura Schulz

Gundula Schulze‐Tanzil

Felix Schumacher

Ismael Secundino

Prosenjit Sen

Roshanak Shams

Bojing Shao

Lei Shi

Toshiyuki Shimizu

Gyu‐Tae Shin

Stepan Shipovskov

Weinian Shou

Noman A Siddiqi

Fernando Simabuco

Dina Costa Simes

Jodie Simpson

Satarudra Prakash Singh

Juliana Smetana

Liudmila Sosulina

James Stowell

Richard Strasser

Young‐Ah Suh

Jianping Sun

Yutaka Suzuki

Erik Swenson

Nobuyuki Takahashi

Josephine Tauer

Mahvash Tavassoli

Yemane Tedros

Daisuke Tezuka

Erica Todd

Kazuaki Tokodai

Nestor V Torres

Eduardo Caio Torres‐Santos

Masashi Toyoda

Jason Treberg

Robert Edwards Treharne

Prosum Tribedi

Timir Tripathi

Chakrapani Tripathi

Madia Trujillo Garre

Hiroki Tsukamoto

Koji Ueda

Mauro Valtieri

Basavaraj Vastrad

Chanabasayya Vastrad

Adrian Velazquez‐Campoy

Elisa Venturi

Adalberto Ramon Vieyra

Susanne Voelkel

Ian Wallace

Chengshu Wang

Jungeng Wang

Yongxia Wang

Yunguan Wang

Xutong Wang

Wei Wang

Ziyan Wang

Komal Waqas

Satoshi Watanabe

Beau Webber

Aidong Wen

Nan‐Ping Weng

Joanna Wesoly

Christian A M Wilson

A Wiszniewska

Stephen G Withers

Matthew Wohlever

Bartosz Wojtas

David W Wood

Yuliang Wu

Ximei Wu

Thomas Wurdinger

Zhuo Xing

Hong‐Tao Xu

Sudhir Kumar Yadav

Sunil Yadav

Masayuki Yamamoto

Motohiro Yamauchi

Bin Yan

Jun Yan

Yueh‐Hsun Kevin Yang

Wentao Yang

Guoyu Yang

Joe Yeong

SC Yeung

Li Yin

Yixing Yuchi

Daniel Załuski

Laura Zannini

Peter G Zaphiropoulos

David Zarkower

Fangqin Zeng

Xian Zhang

Feng Zhang

Li Zhang

ChunYan Zhang

Yanxiao Zhang

Shujian Zhang

Yinlong Zhang

Yunfeng Zhao

Yuanlin Zheng

Huaiping Zheng

Qinjie Zhou

Honggang Zhou

Yuzheng Zhuge

Tali Zitman‐Gal

